# Supplementary material for: A multi-center cross-platform single-cell RNA sequencing reference dataset
Source: Sci Data. 2021 Feb 2;8:39. doi: 10.1038/s41597-021-00809-x (PMC7854649; doi:10.1038/s41597-021-00809-x)
Supplement: Supplementary file 1 [file 41597_2021_809_MOESM1_ESM.pdf]

QC reports

This document is to compare characteristics of features before and after cell filtering. (refer to our manuscript: (https://doi.org/10.1038/s41597-021-00809-x)).

Quality Control

We used four scRNA-seq platforms across four sites, using two well-characterized reference cell lines, a human breast cancer cell line (Sample A) and a matched control ‘normal’ B lymphocyte line (Sample B) derived from the same donor.

Four platforms include:

- 10X Genomics Chromium
- Fluidigm C1
- Fluidigm C1 HT
- Takara Bio ICELL8

Four sites include:

- Loma Linda University (LLU)
- National Cancer Institute (NCI)
- US Food and Drug Administration (FDA)
- Takara Bio USA (TBU)

Overall, we generated 20 scRNA-seq datasets, including 3´-transcript and full-length transcript scRNA-seq datasets. We sequenced a total of 49793 single cells with either 3´ or full-length scRNA-seq methods before filtering.

Cell number of each datasets before filtering:

```
table(df_pre$dataset)
```

```
## 
##        10X_LLU_A          10X_NCI_A        10X_NCI_M_A        C1_FDA_HT_A 
##             3045               6425               6483                203 
##         C1_LLU_A        ICELL8_SE_A        ICELL8_PE_A          10X_LLU_B 
##               80                598                600               1439 
##        10X_NCI_B        10X_NCI_M_B        C1_FDA_HT_B           C1_LLU_B 
##             3296               3273                241                 66 
##      ICELL8_SE_B        ICELL8_PE_B      10X_LLU_Mix10        10X_NCI_Mix5 
##              596                600               4082               4060 
##    10X_NCI_Mix5_F    10X_NCI_M_Mix5  10X_NCI_M_Mix5_F 10X_NCI_M_Mix5_F2 
##             4234               4028               4122               2322 
```

QC plot before filtering

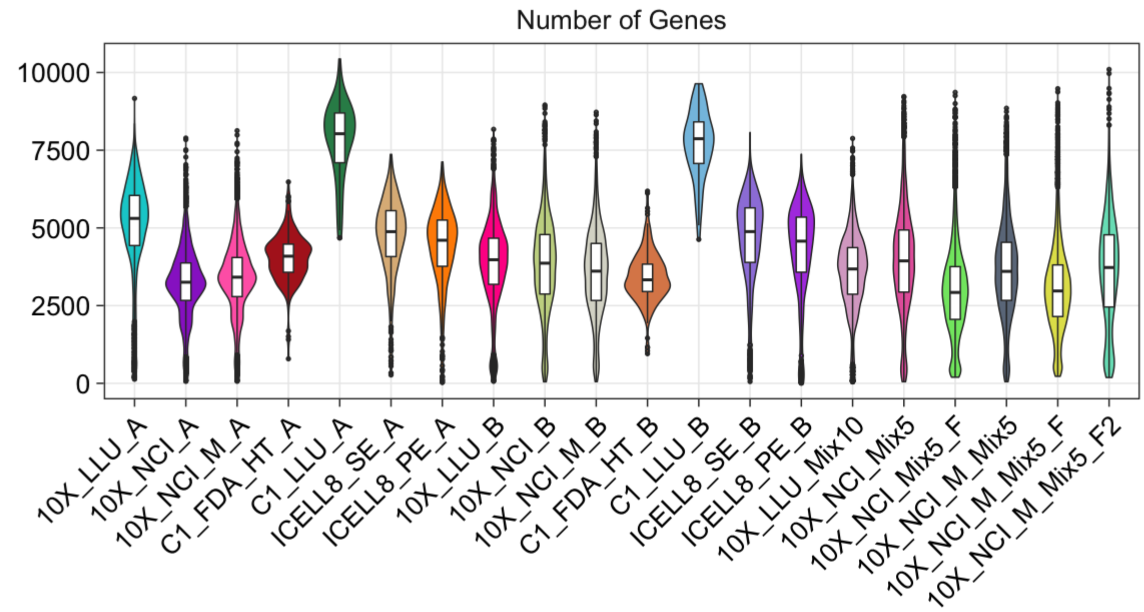

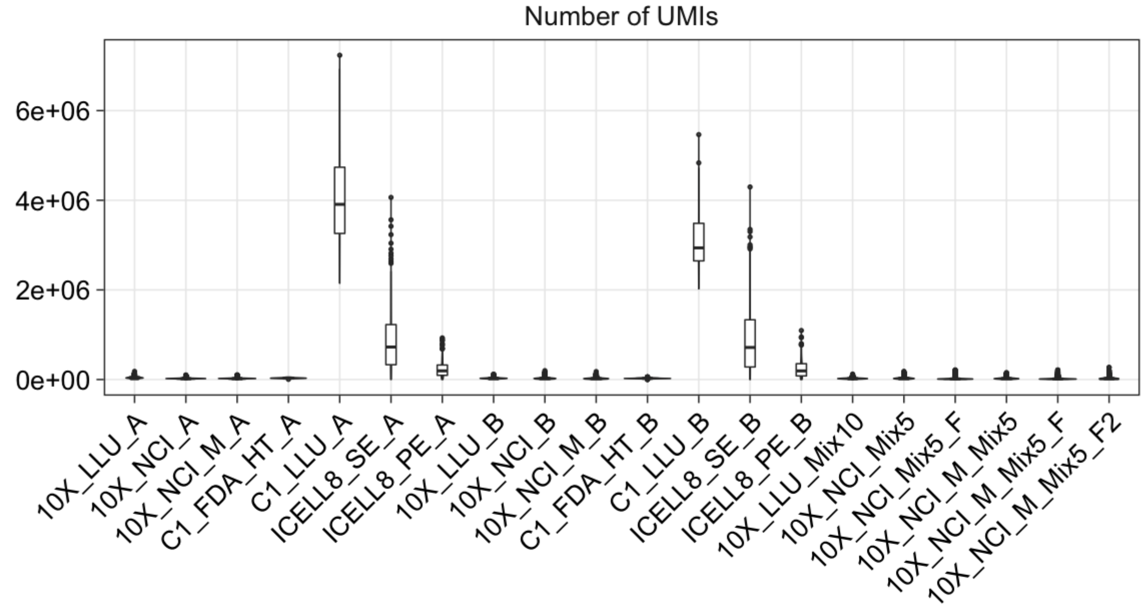

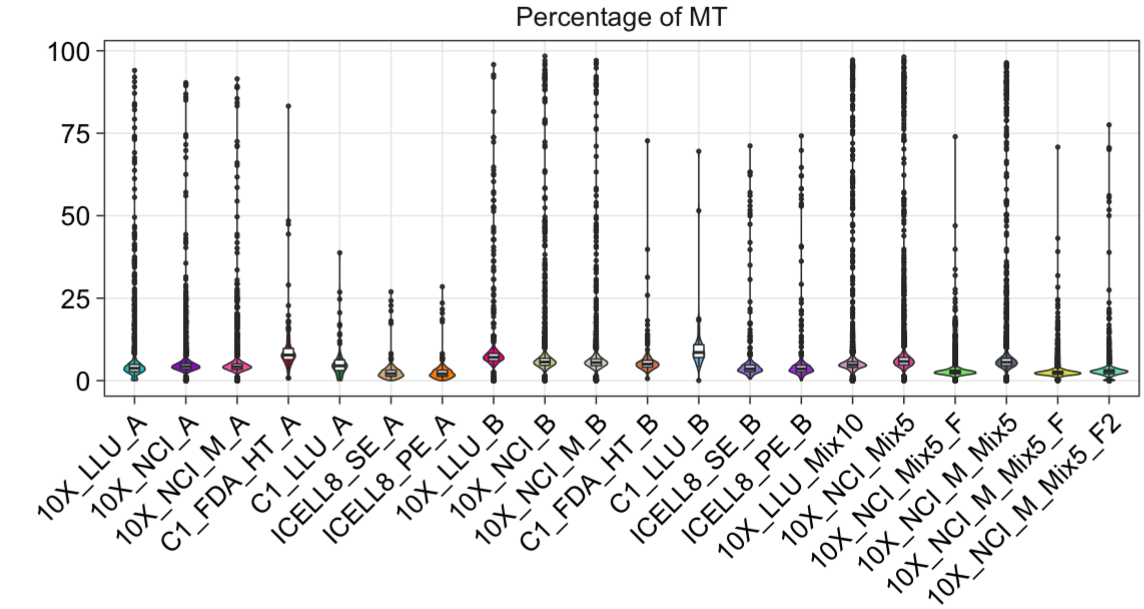

Feature-feature relationships (before filtering)

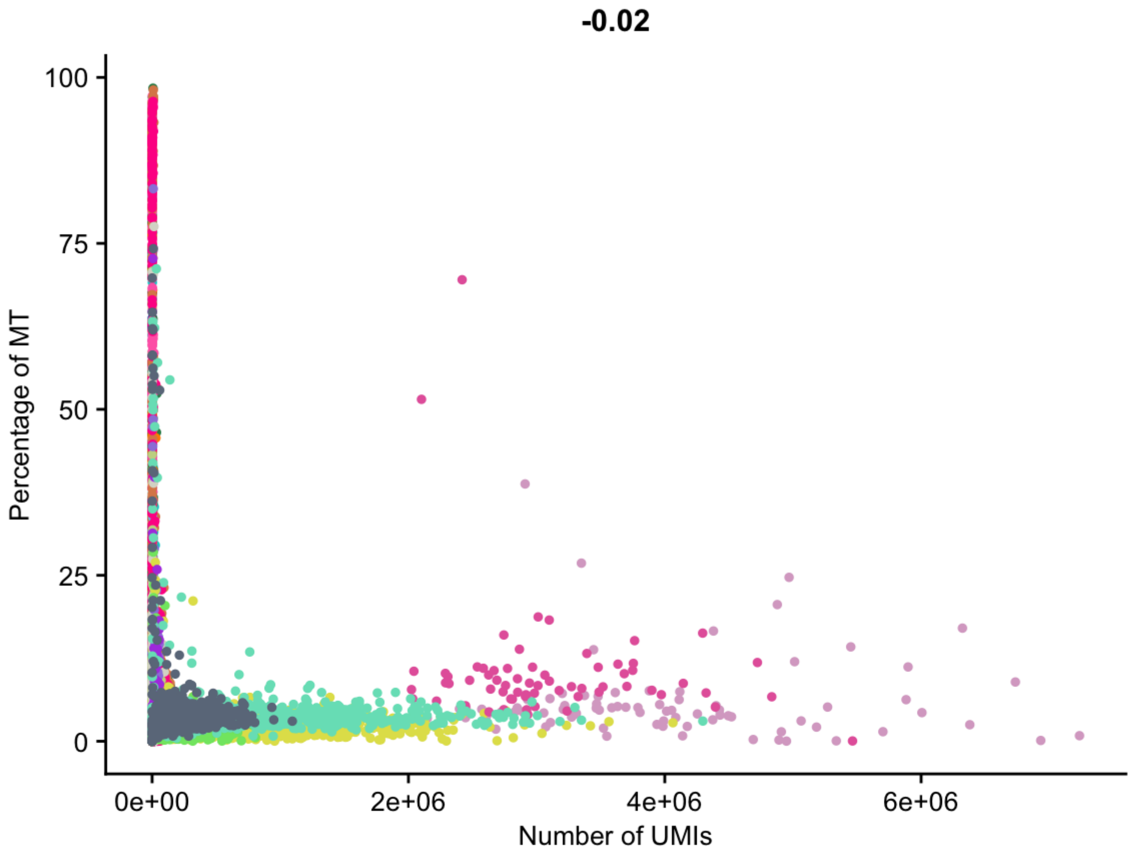

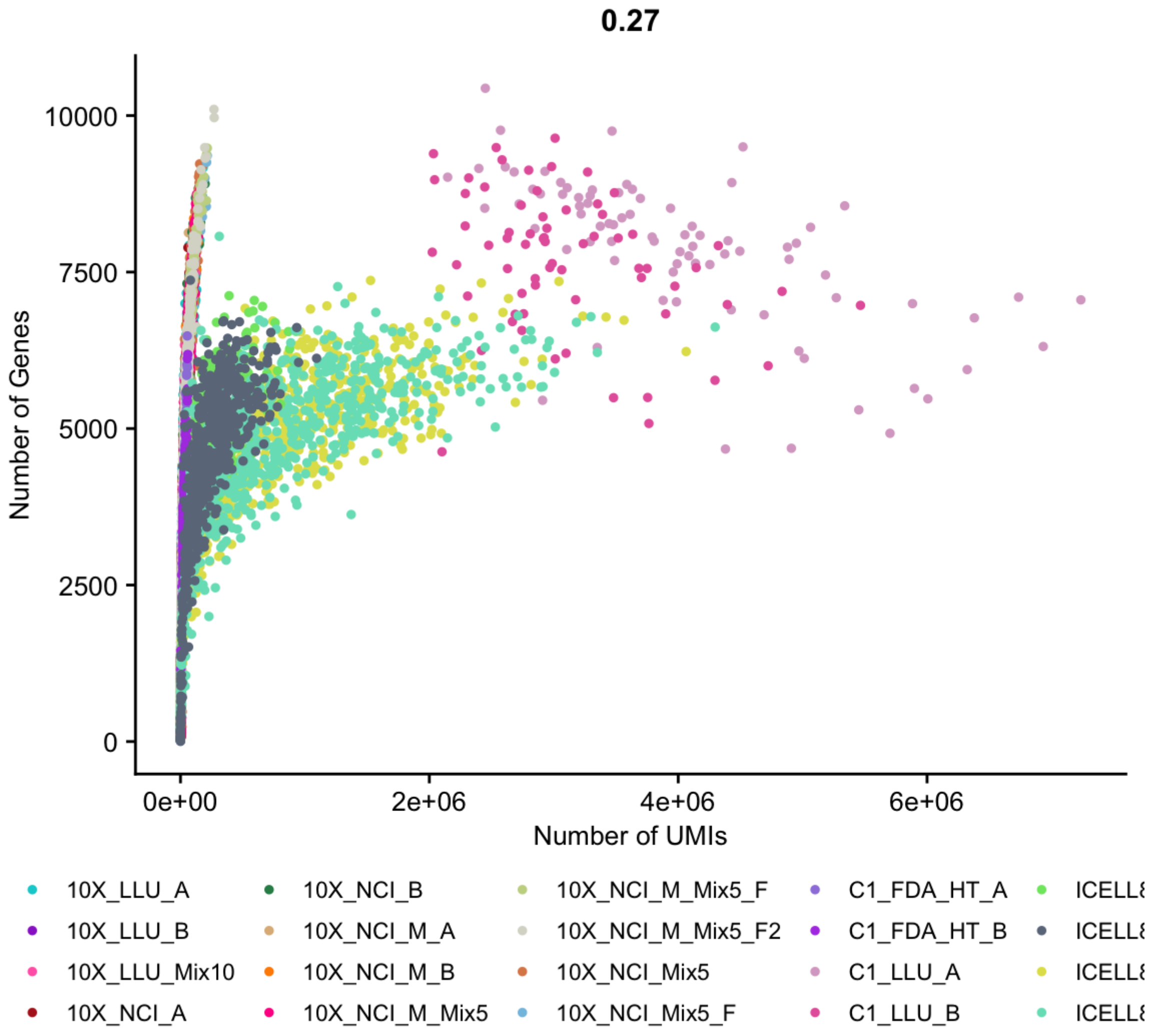

Linear & non-linear dimensional reduction (before filtering)

```
Cells <- Cells %>%
  NormalizeData() %>%
  FindVariableFeatures(selection.method = "vst", nfeatures = 2000) %>%
  ScaleData()
Cells <- RunPCA(Cells, features = VariableFeatures(object = Cells))
print(Cells[["pca"]], dims = 1:6, nfeatures = 5)
```

```
## PC_ 1
## Positive:  ENSG00000150093, ENSG00000105974, ENSG00000197956, ENSG00000197747, ENSG000001827
18
## Negative:  ENSG00000019582, ENSG00000204287, ENSG00000205542, ENSG00000162511, ENSG000001113
48
## PC_ 2
## Positive:  ENSG00000131669, ENSG00000082014, ENSG00000197956, ENSG00000095739, ENSG000000872
45
## Negative:  ENSG00000139644, ENSG00000110955, ENSG00000182481, ENSG00000150753, ENSG000001964
19
## PC_ 3
## Positive:  ENSG00000206075, ENSG00000140105, ENSG00000163993, ENSG00000070669, ENSG000001068
23
## Negative:  ENSG00000101057, ENSG00000066279, ENSG00000198938, ENSG00000198763, ENSG000001988
99
## PC_ 4
## Positive:  ENSG00000188229, ENSG00000111640, ENSG00000077152, ENSG00000143933, ENSG000001641
04
## Negative:  ENSG00000198886, ENSG00000198786, ENSG00000198804, ENSG00000198899, ENSG000001987
12
## PC_ 5
## Positive:  ENSG00000152234, ENSG00000108679, ENSG00000087245, ENSG00000182326, ENSG000000957
39
## Negative:  ENSG00000175592, ENSG00000134668, ENSG00000107984, ENSG00000163814, ENSG000000706
69
## PC_ 6
## Positive:  ENSG00000131747, ENSG00000100526, ENSG00000066279, ENSG00000198901, ENSG000001387
78
## Negative:  ENSG00000136156, ENSG00000101608, ENSG00000134668, ENSG00000096384, ENSG000001009
06
```

```
VizDimLoadings(Cells, dims = 1:2, reduction = "pca")
```

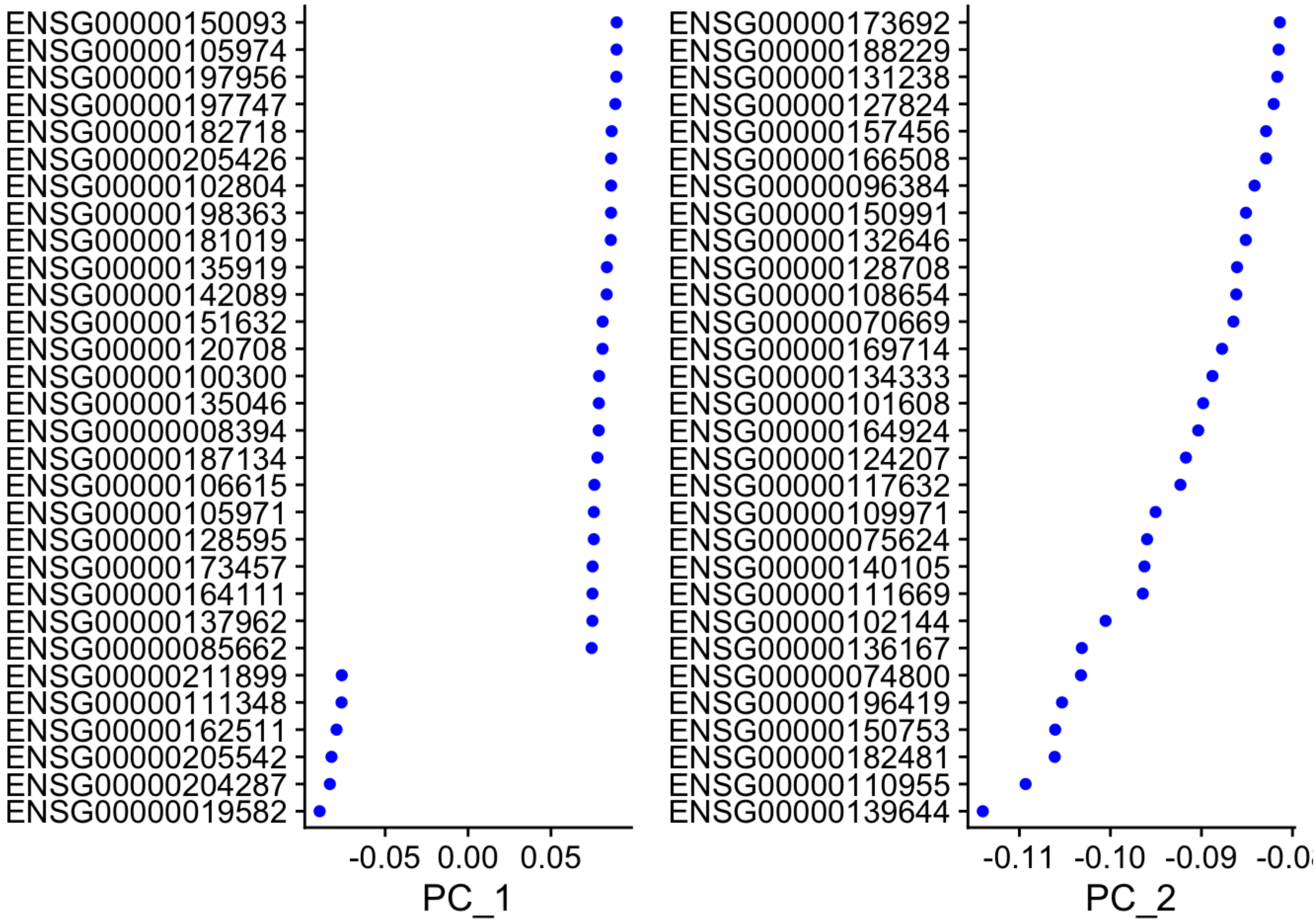


```
DimPlot(Cells, reduction = "pca", group.by = "dataset")
```

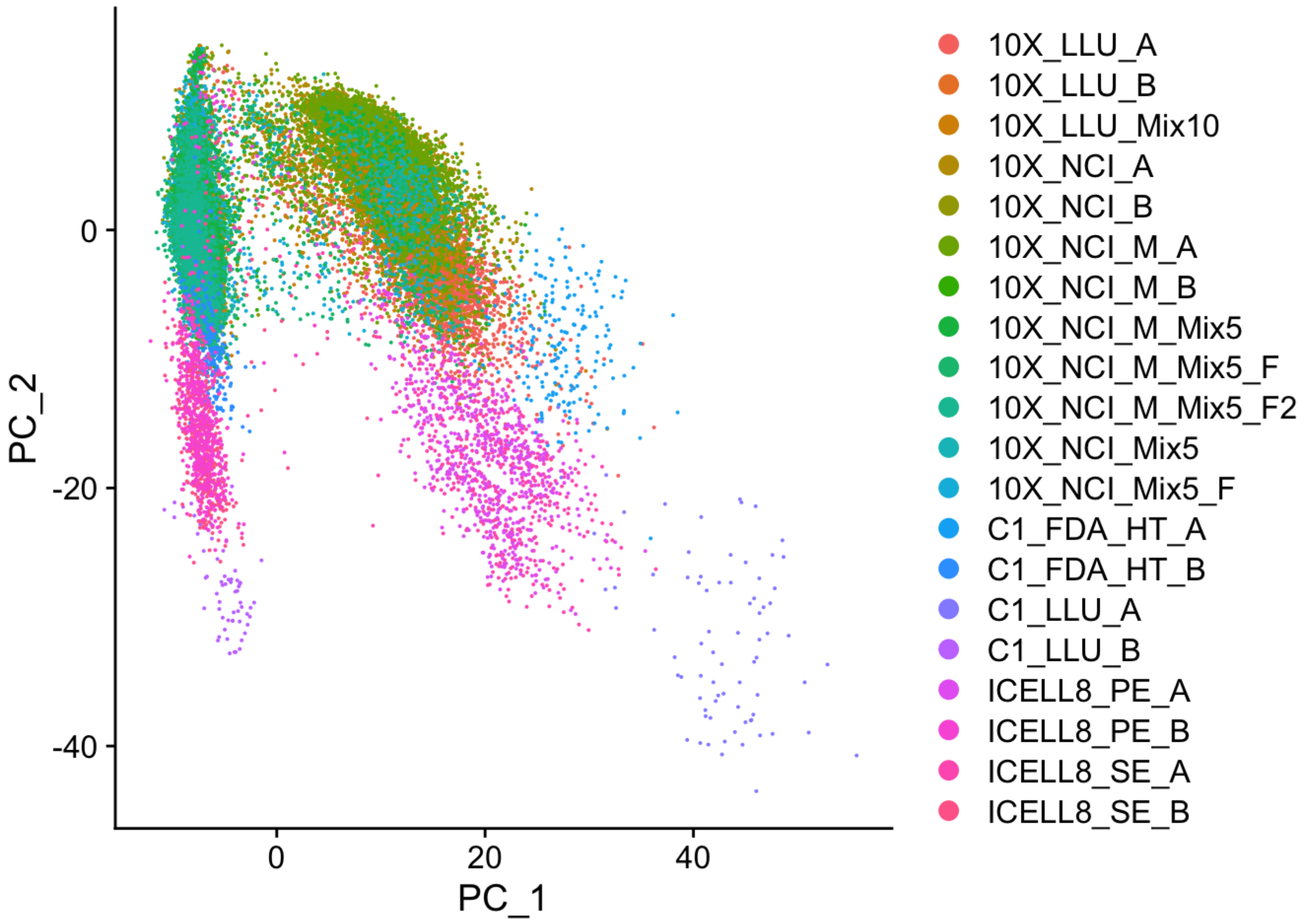


```
DimHeatmap(Cells, dims = 1:6, cells = 500, balanced = TRUE)
```

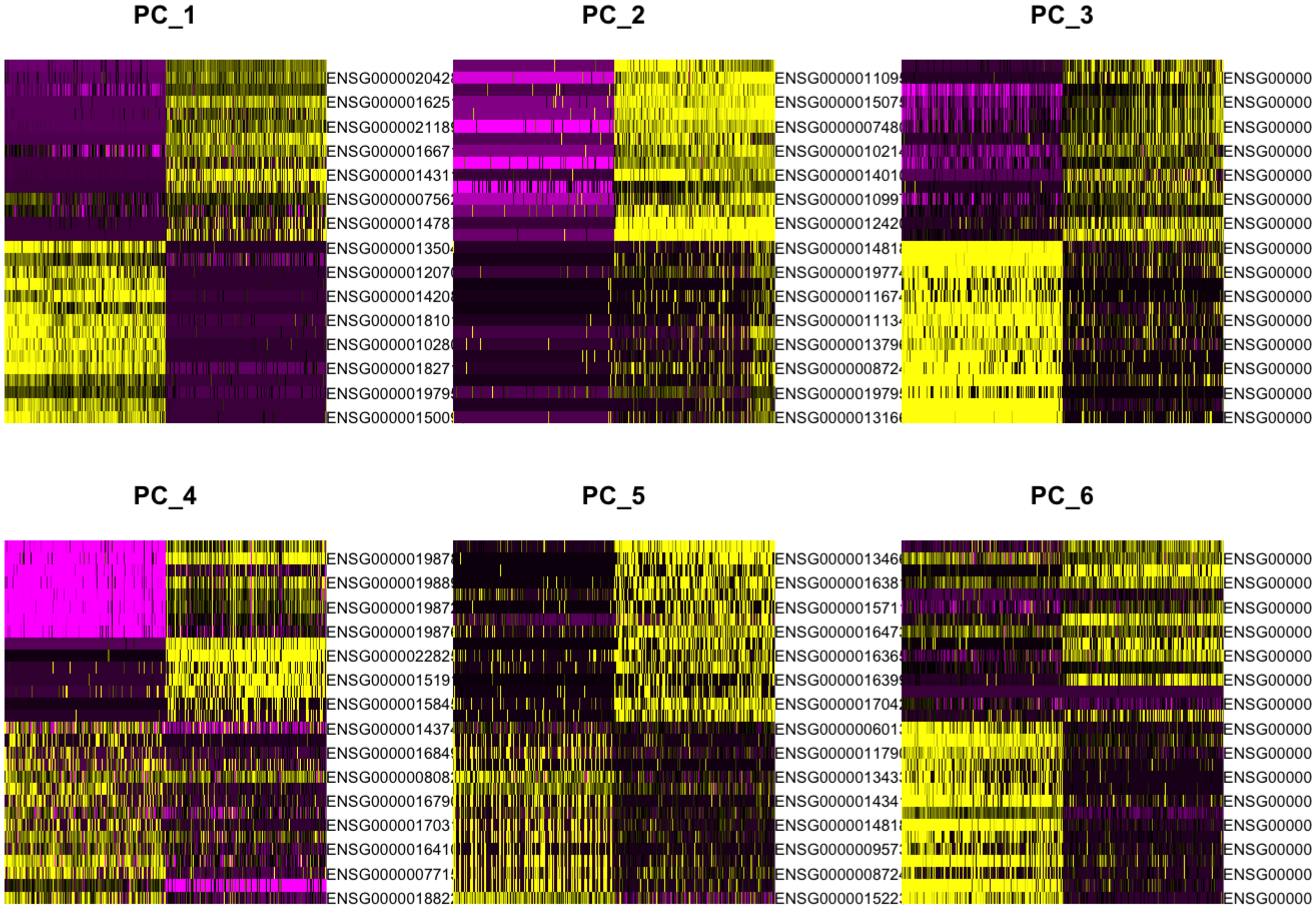


```
ElbowPlot(Cells)
```

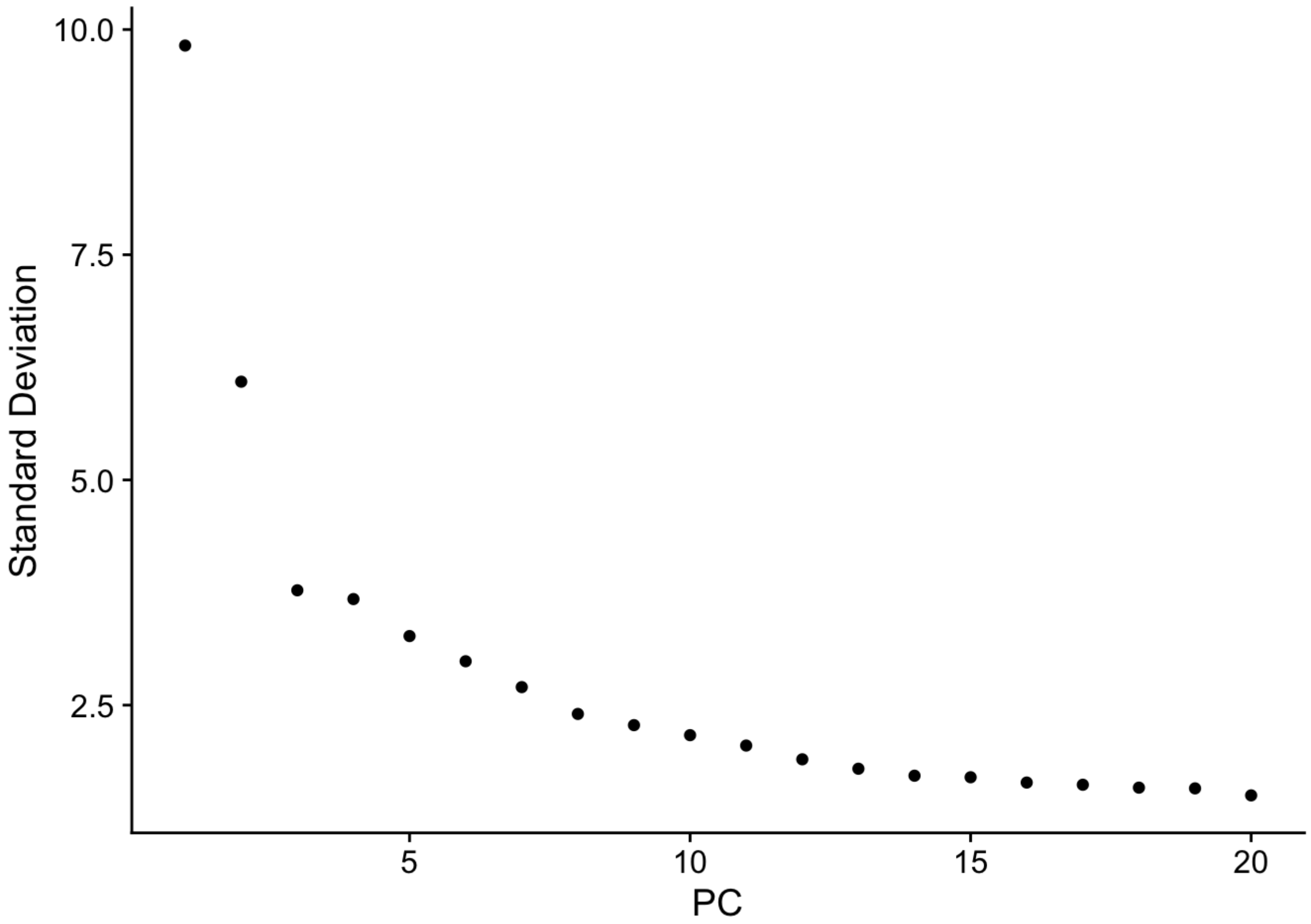

```
Cells <- RunUMAP(Cells, dims = 1:15)
DimPlot(Cells, reduction = "umap", group.by = "dataset") +
  theme(text = element_text(size = 10),
                axis.text = element_text(size = 10),
                title= element_text(size = 10)) +
  theme(legend.position = "bottom")
```

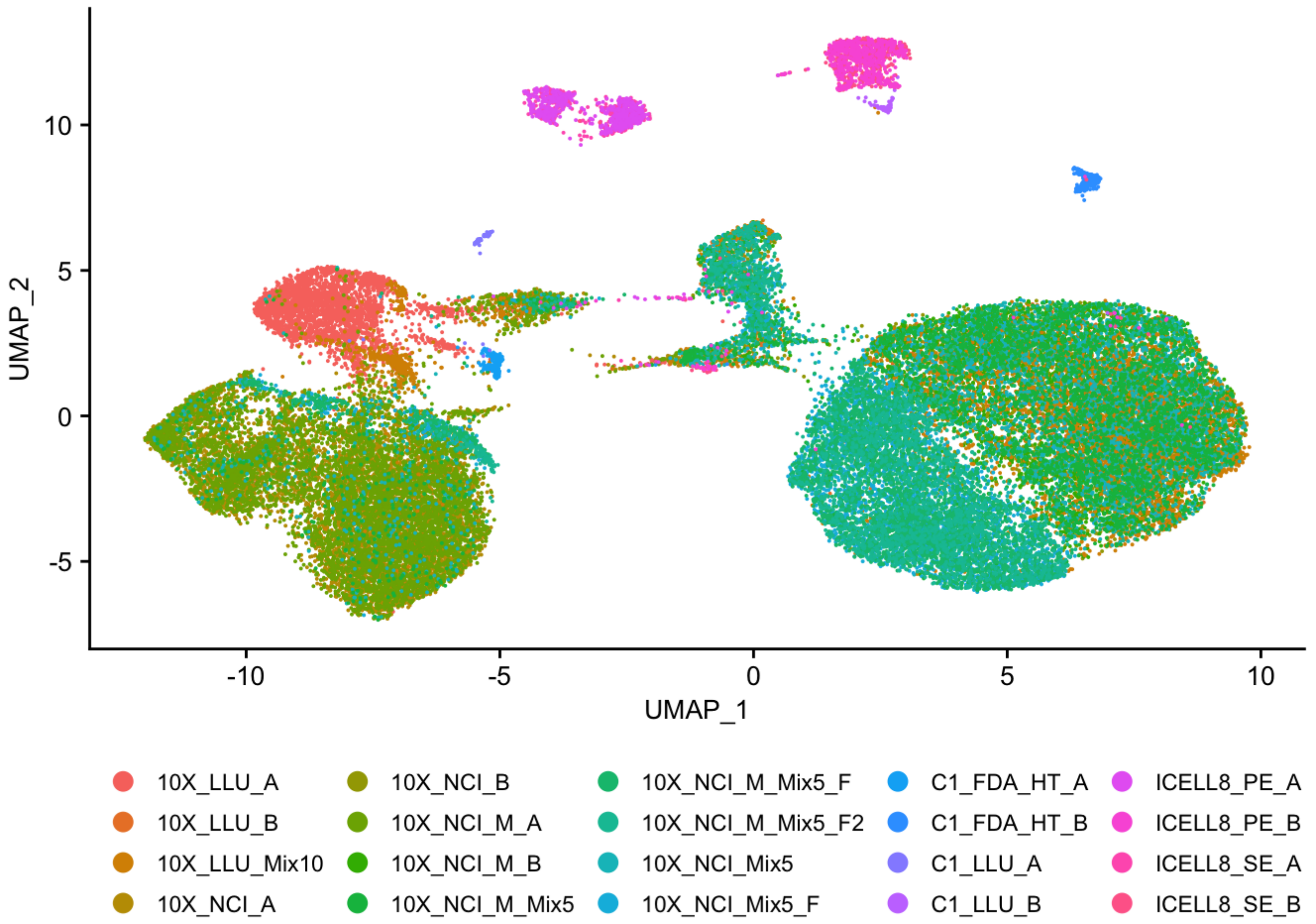

Filtering

Cells were filtered in three steps:

- First, we filter cells using the CreateSeuratObject function of Seurat package (version 3.1.0) using options: min.cells = 3, min.features = 200.
- Second, cells were filtered with mitochondrial (Mito) gene regression >10%.
- Third, cells were filtered with [10^(mean(log10(Total_mRNAs)), 2*sd(log10(Total_mRNAs))].

QC plot after filtering

After filtering, a total of 45335 single cells passed. Cell number of each datasets after filtering:

```
table(df_post$dataset)
```

```
##
##        10X_LLU_A         10X_NCI_A       10X_NCI_M_A       C1_FDA_HT_A
##             2735              5996              6035               155
##         C1_LLU_A       ICELL8_SE_A       ICELL8_PE_A         10X_LLU_B
##               63               569               566              1267
##        10X_NCI_B       10X_NCI_M_B       C1_FDA_HT_B          C1_LLU_B
##             2945              2953               220                42
##      ICELL8_SE_B       ICELL8_PE_B      10X_LLU_Mix10      10X_NCI_Mix5
##              538               537              3785              3601
##    10X_NCI_Mix5_F    10X_NCI_M_Mix5  10X_NCI_M_Mix5_F 10X_NCI_M_Mix5_F2
##             3830              3622              3772              2104
```

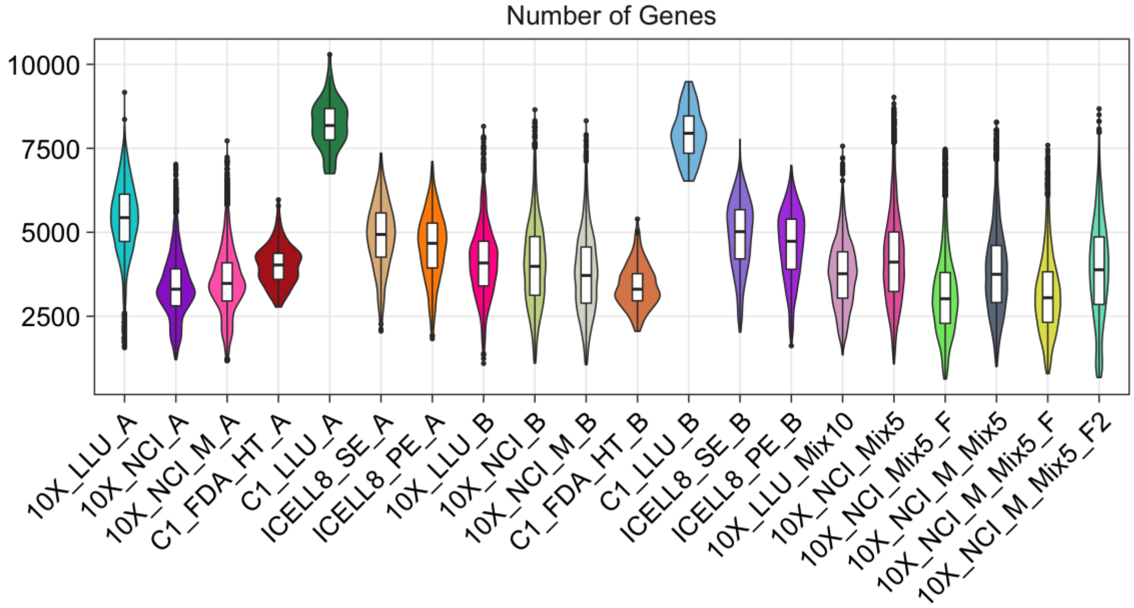

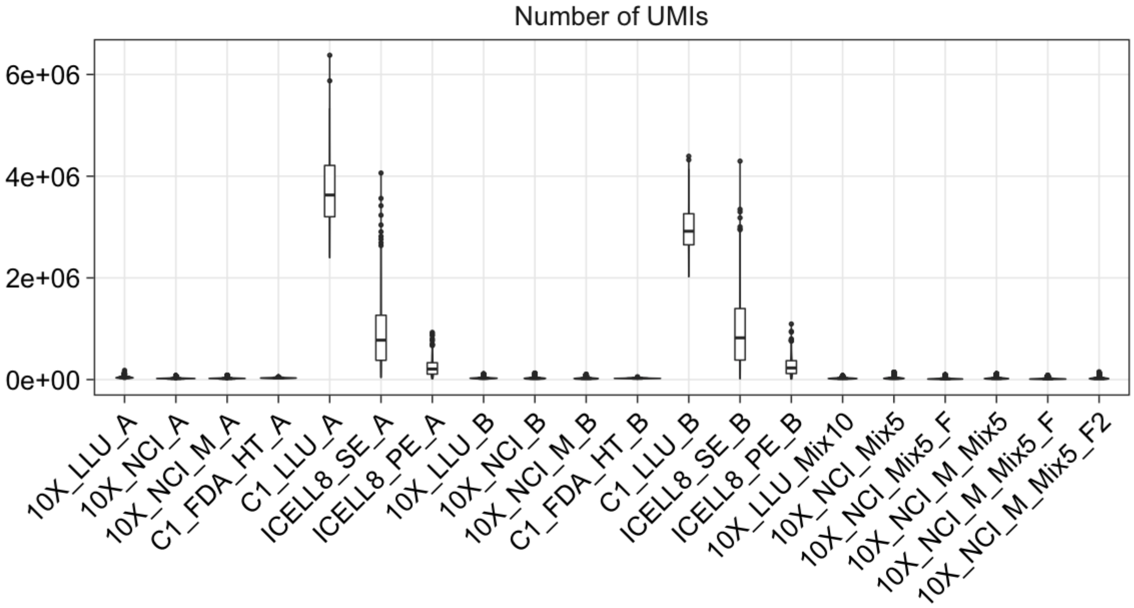

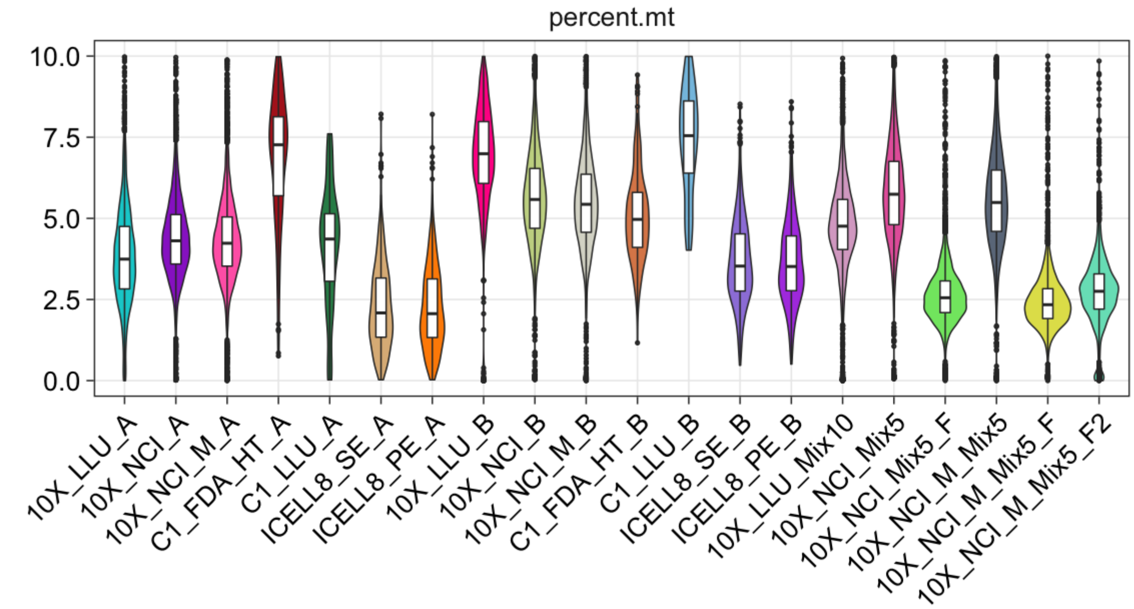

Feature-feature relationships (after filtering)

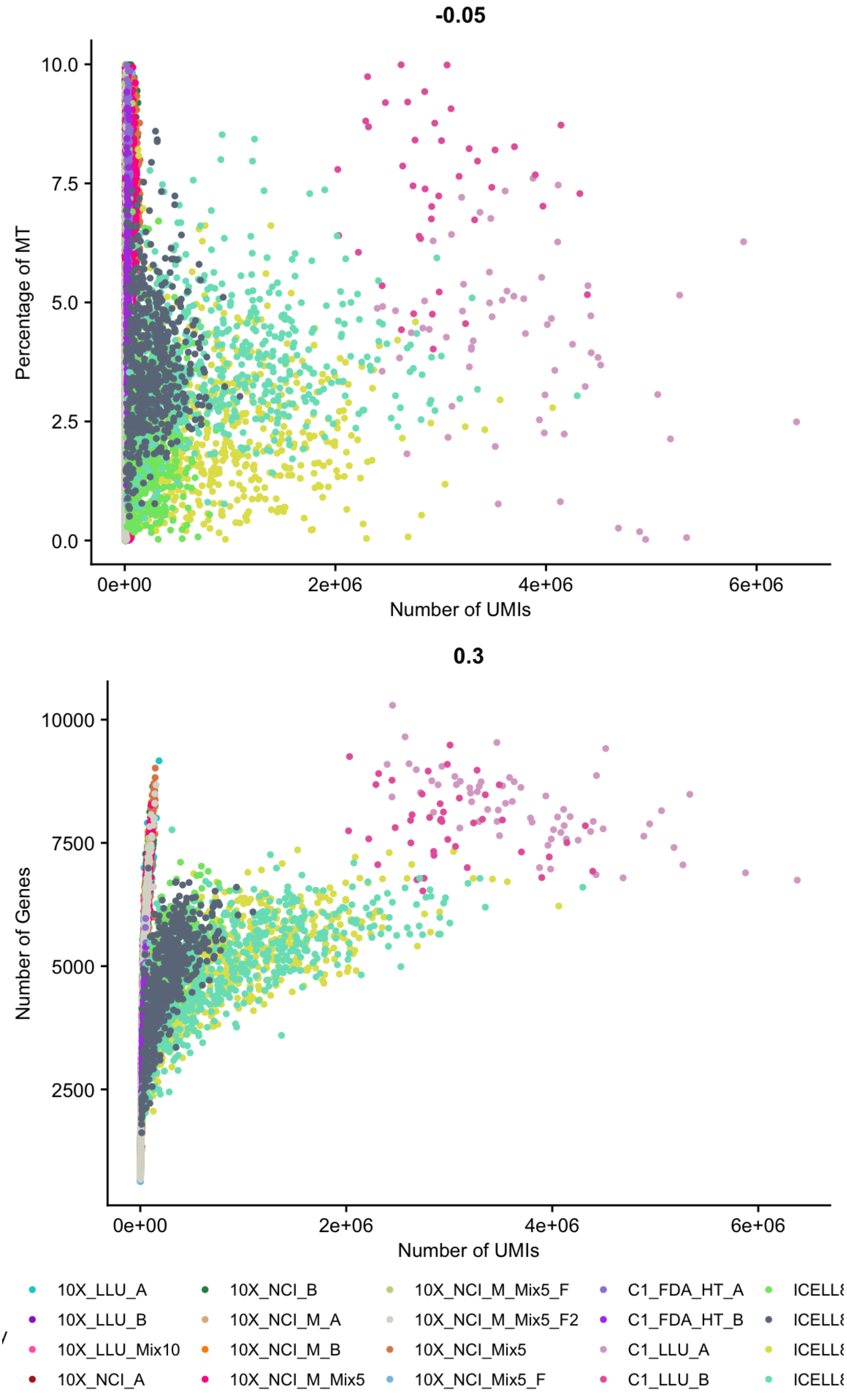

Linear & non-linear dimensional reduction (after filtering)

```
Cells <- Cells %>%
  NormalizeData() %>%
  FindVariableFeatures(selection.method = "vst", nfeatures = 2000) %>%
  ScaleData()
Cells <- RunPCA(Cells, features = VariableFeatures(object = Cells))
print(Cells[["pca"]], dims = 1:6, nfeatures = 5)
```

```
## PC_ 1
## Positive:  ENSG00000197956, ENSG00000197747, ENSG00000105974, ENSG00000150093, ENSG000001810
19
## Negative:  ENSG00000019582, ENSG00000204287, ENSG00000205542, ENSG00000162511, ENSG000001113
48
## PC_ 2
## Positive:  ENSG00000139644, ENSG00000110955, ENSG00000150753, ENSG00000196419, ENSG000001824
81
## Negative:  ENSG00000117906, ENSG00000131669, ENSG00000182253, ENSG00000112335, ENSG000000957
39
## PC_ 3
## Positive:  ENSG00000179091, ENSG00000080824, ENSG00000117632, ENSG00000131747, ENSG000001882
29
## Negative:  ENSG00000206075, ENSG00000140105, ENSG00000100439, ENSG00000163993, ENSG000001703
45
## PC_ 4
## Positive:  ENSG00000175592, ENSG00000101255, ENSG00000107984, ENSG00000115756, ENSG000001324
65
## Negative:  ENSG00000152234, ENSG00000270550, ENSG00000182326, ENSG00000211669, ENSG000000672
25
## PC_ 5
## Positive:  ENSG00000198786, ENSG00000198886, ENSG00000198804, ENSG00000198712, ENSG000001987
27
## Negative:  ENSG00000111640, ENSG00000141543, ENSG00000188229, ENSG00000150991, ENSG000001439
33
## PC_ 6
## Positive:  ENSG00000096384, ENSG00000100906, ENSG00000107984, ENSG00000157111, ENSG000000756
24
## Negative:  ENSG00000131747, ENSG00000087586, ENSG00000166851, ENSG00000100526, ENSG000001176
50
```

```
VizDimLoadings(Cells, dims = 1:2, reduction = "pca")
```

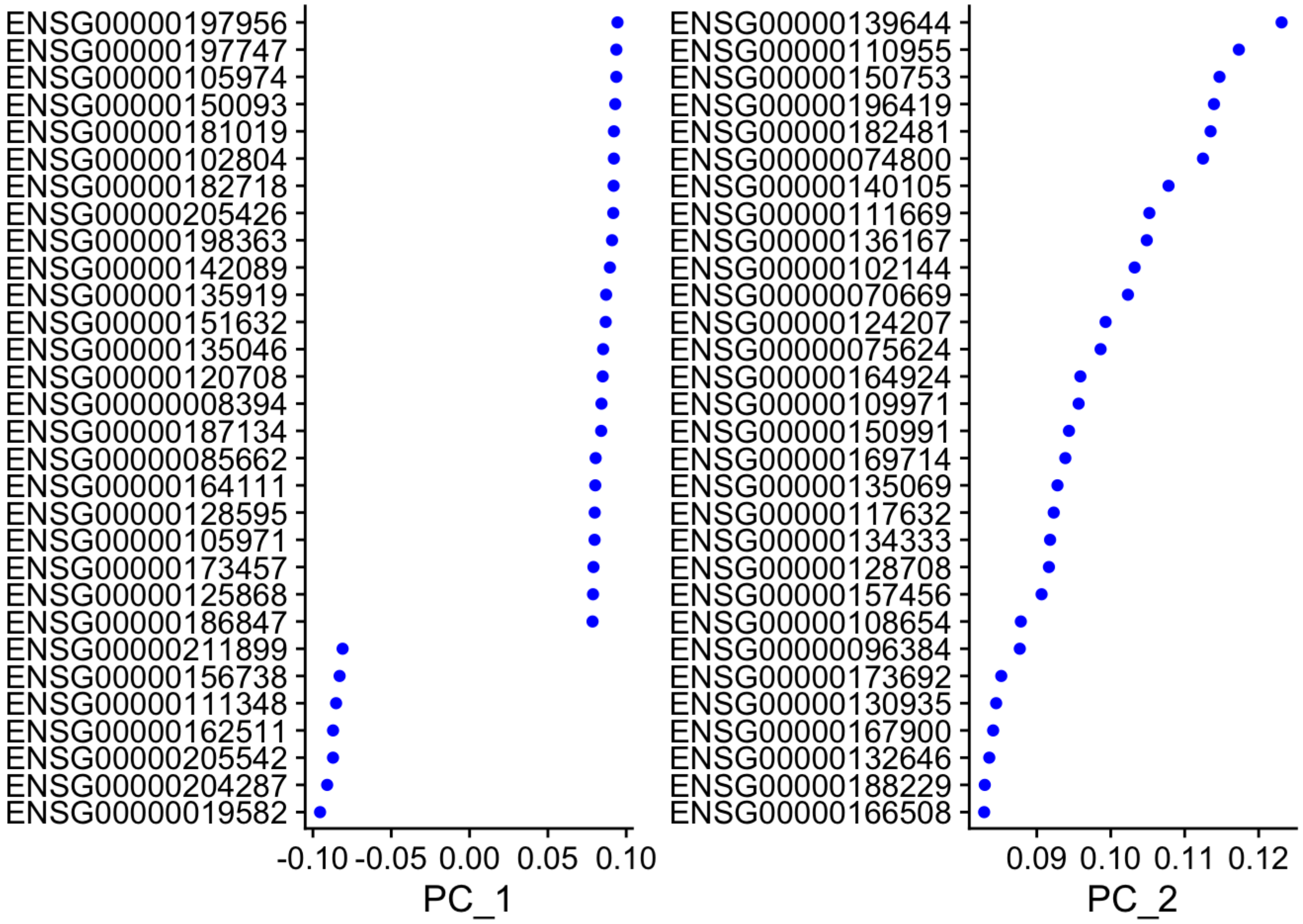


```
DimPlot(Cells, reduction = "pca", group.by = "dataset")
```

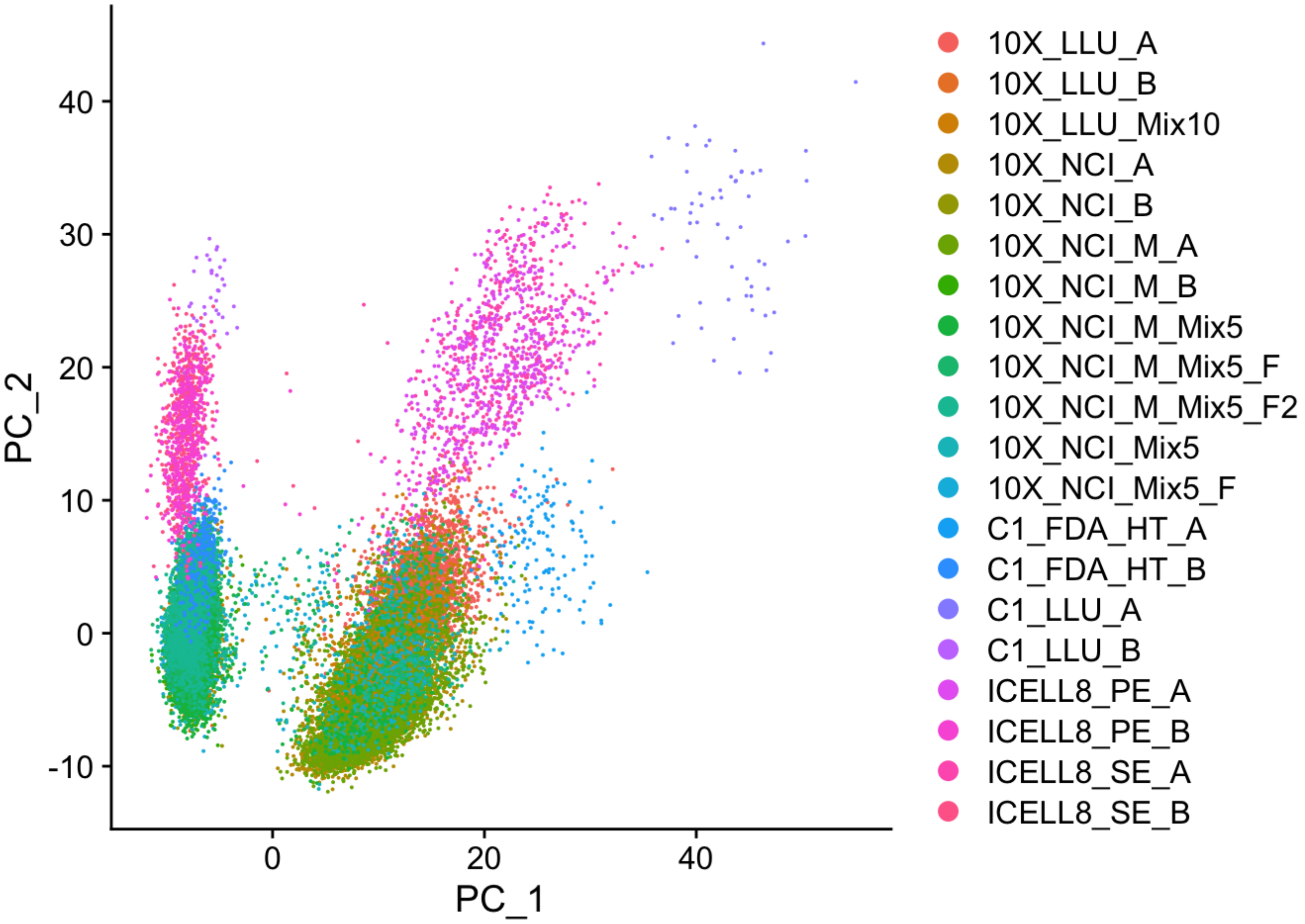


```
DimHeatmap(Cells, dims = 1:6, cells = 500, balanced = TRUE)
```

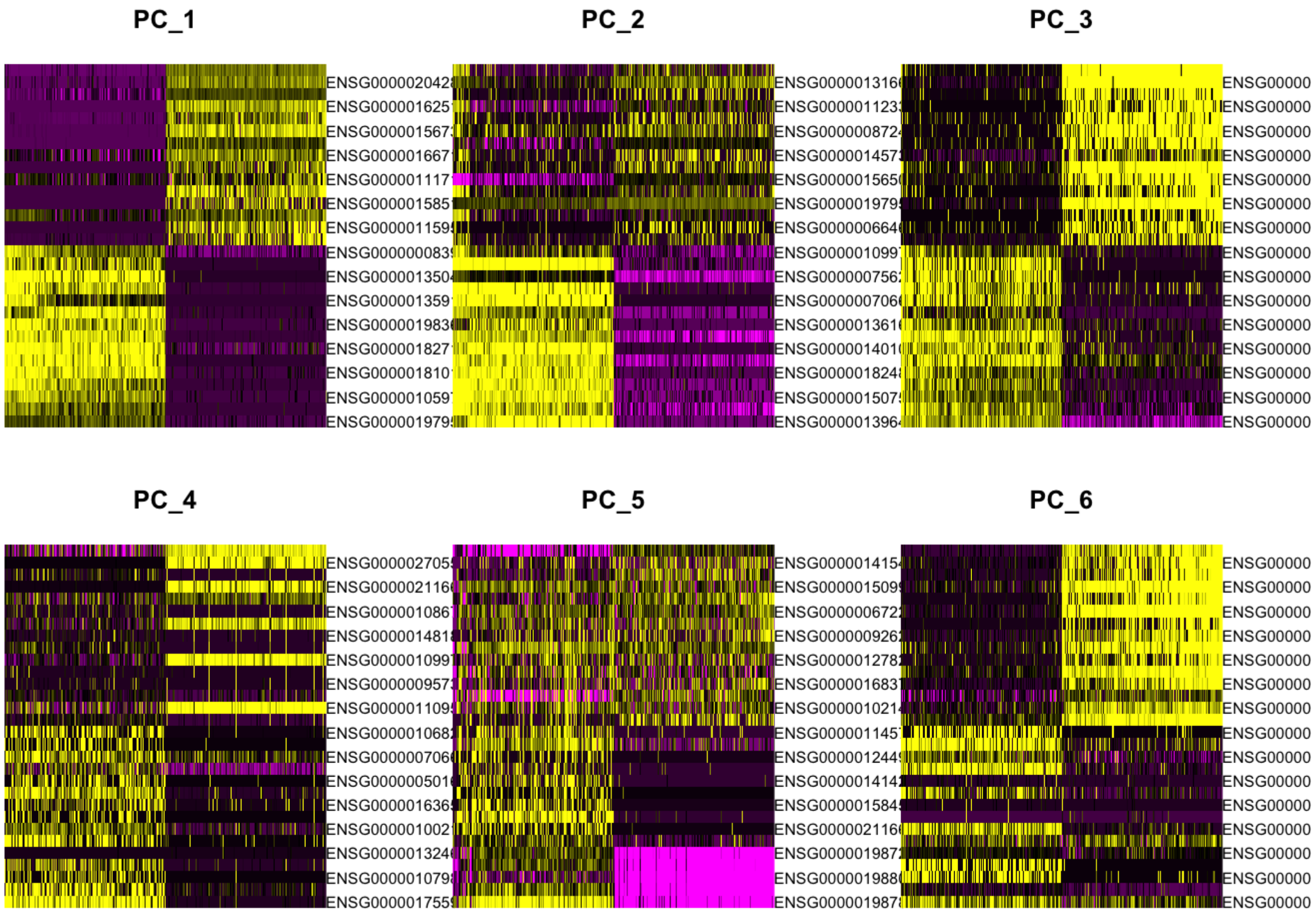


```
ElbowPlot(Cells)
```

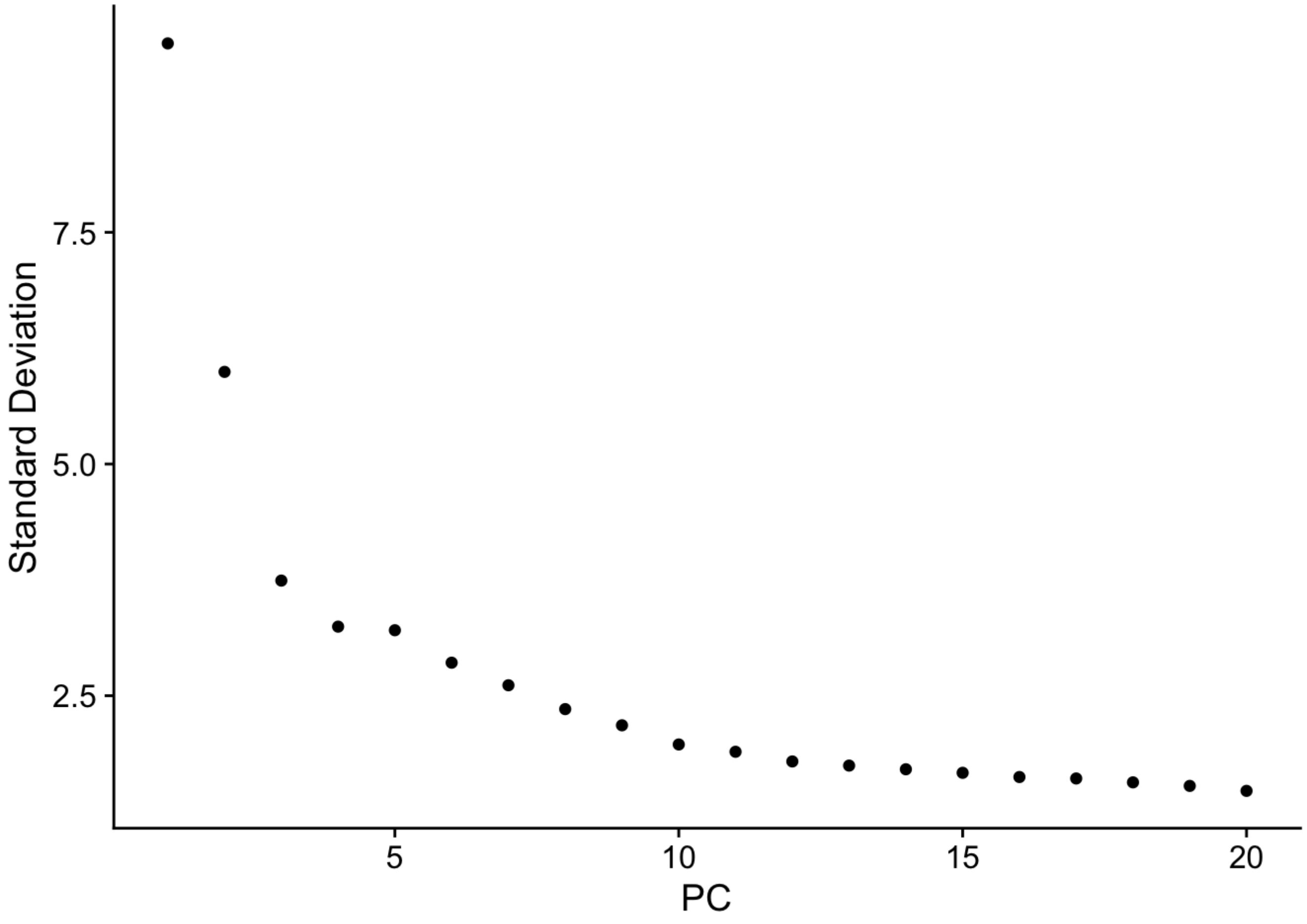

```
Cells <- RunUMAP(Cells, dims = 1:15)
DimPlot(Cells, reduction = "umap", group.by = "dataset") +
  theme(text = element_text(size = 10),
               axis.text = element_text(size = 10),
               title= element_text(size = 10)) +
  theme(legend.position = "bottom")
```

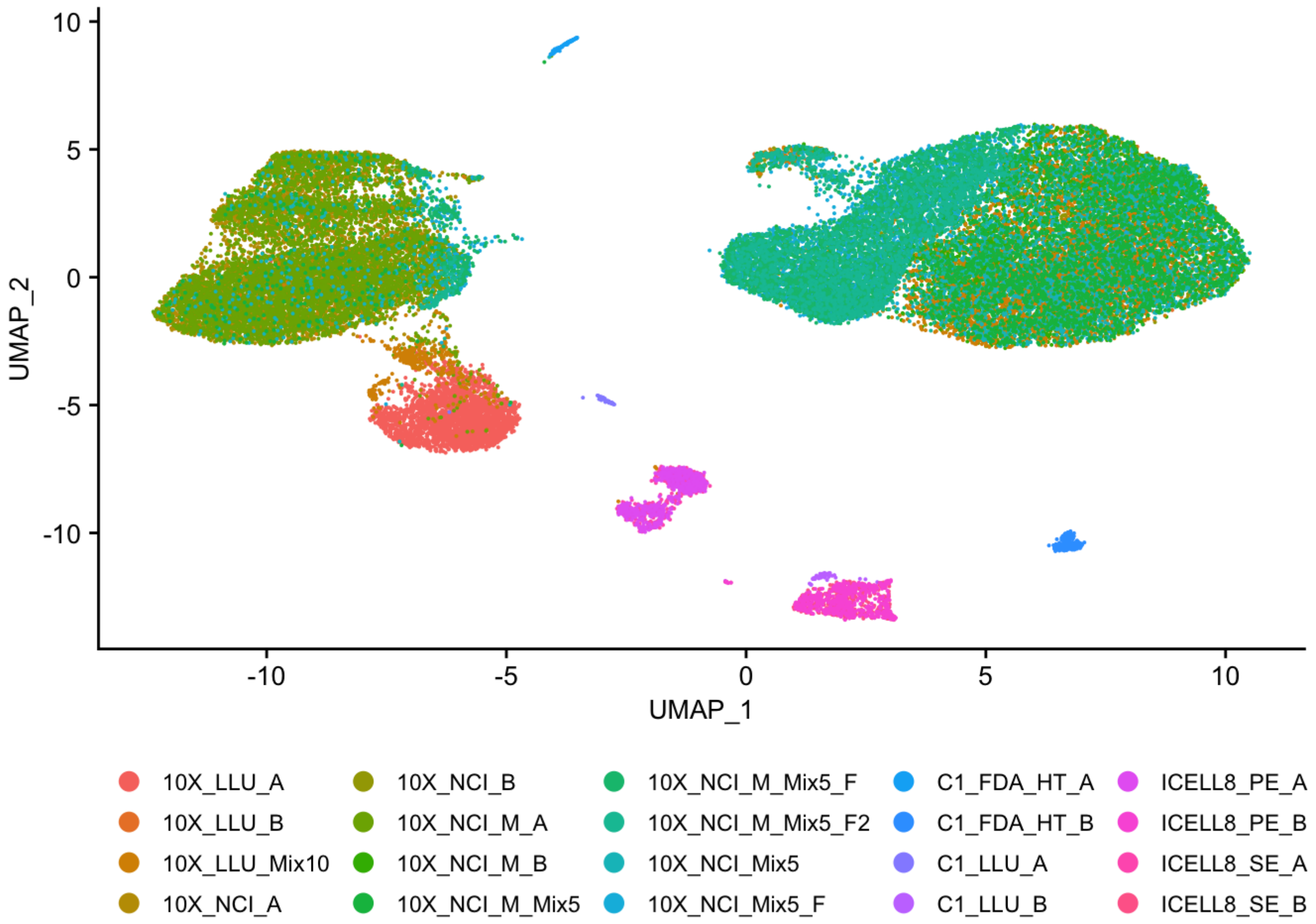

Summary

A total of 4458 cells were removed according to the filtering criteria:

```
table(df_pre$dataset) - table(df_post$dataset)
```

```
##
##        10X_LLU_A         10X_NCI_A        10X_NCI_M_A        C1_FDA_HT_A
##              310               429                448                 48
##         C1_LLU_A       ICELL8_SE_A        ICELL8_PE_A          10X_LLU_B
##               17                29                 34                172
##        10X_NCI_B       10X_NCI_M_B        C1_FDA_HT_B           C1_LLU_B
##              351               320                 21                 24
##      ICELL8_SE_B       ICELL8_PE_B      10X_LLU_Mix10        10X_NCI_Mix5
##               58                63                297                459
##     10X_NCI_Mix5_F    10X_NCI_M_Mix5  10X_NCI_M_Mix5_F 10X_NCI_M_Mix5_F2
##              404               406                350                218
```

```
sessionInfo()
```

```
## R version 3.6.0 (2019-04-26)
## Platform: x86_64-apple-darwin15.6.0 (64-bit)
## Running under: macOS  10.15.7
##
## Matrix products: default
## BLAS:   /Library/Frameworks/R.framework/Versions/3.6/Resources/lib/libRblas.0.dylib
## LAPACK: /Library/Frameworks/R.framework/Versions/3.6/Resources/lib/libRlapack.dylib
##
## locale:
## [1] en_US.UTF-8/en_US.UTF-8/en_US.UTF-8/C/en_US.UTF-8/en_US.UTF-8
##
## attached base packages:
## [1] stats     graphics  grDevices utils     datasets  methods   base
##
## other attached packages:
## [1] ggpubr_0.2.3  magrittr_1.5  ggplot2_3.3.2 plyr_1.8.4    tidyr_1.0.0
## [6] Seurat_3.1.0
##
## loaded via a namespace (and not attached):
##  [1] tsne_0.1-3          nlme_3.1-139        bitops_1.0-6
##  [4] RcppAnnoy_0.0.13    RColorBrewer_1.1-2  httr_1.4.1
##  [7] sctransform_0.2.0   tools_3.6.0         R6_2.4.0
## [10] irlba_2.3.3         KernSmooth_2.23-15  uwot_0.1.4
## [13] lazyeval_0.2.2      colorspace_1.4-1    withr_2.1.2
## [16] npsurv_0.4-0        tidyselect_1.1.0    gridExtra_2.3
## [19] compiler_3.6.0      plotly_4.9.0        labeling_0.3
## [22] caTools_1.17.1.2    scales_1.1.1        lmtest_0.9-37
## [25] ggridges_0.5.1      pbapply_1.4-2       stringr_1.4.0
## [28] digest_0.6.21       rmarkdown_2.1       R.utils_2.9.0
## [31] pkgconfig_2.0.3     htmltools_0.5.0     bibtex_0.4.2
## [34] htmlwidgets_1.3     rlang_0.4.6         farver_2.0.3
## [37] generics_0.0.2      zoo_1.8-6           jsonlite_1.6
## [40] ica_1.0-2           gtools_3.8.1        dplyr_1.0.0
## [43] R.oo_1.22.0         Matrix_1.2-17       Rcpp_1.0.2
## [46] munsell_0.5.0       ape_5.3             reticulate_1.13
## [49] lifecycle_0.2.0     R.methodsS3_1.7.1   stringi_1.4.3
## [52] yaml_2.2.0          gbRd_0.4-11         MASS_7.3-51.4
## [55] gplots_3.0.1.1      Rtsne_0.15          grid_3.6.0
## [58] parallel_3.6.0      gdata_2.18.0        listenv_0.7.0
## [61] ggrepel_0.8.1       crayon_1.3.4        lattice_0.20-38
## [64] cowplot_1.0.0       splines_3.6.0       SDMTools_1.1-221.1
## [67] knitr_1.25          pillar_1.4.4        igraph_1.2.4.1
## [70] ggsignif_0.6.0      future.apply_1.3.0  reshape2_1.4.3
## [73] codetools_0.2-16    leiden_0.3.1        glue_1.4.1
## [76] evaluate_0.14       lsei_1.2-0          metap_1.1
## [79] RcppParallel_4.4.4  data.table_1.12.2   vctrs_0.3.1
## [82] png_0.1-7           Rdpack_0.11-0       gtable_0.3.0
## [85] RANN_2.6.1          purrr_0.3.2         future_1.14.0
## [88] xfun_0.10           rsvd_1.0.2          RSpectra_0.15-0
## [91] survival_3.1-11     viridisLite_0.3.0   tibble_3.0.1
## [94] cluster_2.0.8       globals_0.12.4      fitdistrplus_1.0-14
## [97] ellipsis_0.3.0      ROCR_1.0-7
```
